# Coupling analysis of crane accident risks based on Bayesian network and the N-K model

**DOI:** 10.1038/s41598-024-51425-9

**Published:** 2024-01-11

**Authors:** Bang-Jie Wu, Liang-Hai Jin, Xia-Zhong Zheng, Shu Chen

**Affiliations:** 1 Hubei Key Laboratory of Construction and Management in Hydropower Engineering, China Three Gorges University, Yichang, 443002 China; 2 College of Mechanical and Power Engineering, China Three Gorges University, Yichang, 443002 China; 3 College of Hydraulic and Environmental Engineering, China Three Gorges University, Yichang, 443002 China

**Keywords:** Engineering, Civil engineering

## Abstract

Crane usage is pervasive on construction sites, however, it is associated with a notably high accident rate. The analyzing of crane accident risks is essential for accident prevention, control, and ensuring the safety of lifting operations. Hence, significant emphasis should be placed on understanding the interaction among various risk factors. This paper proposes a quantitative coupling method for human, machine, management, and environmental risk factors in crane accidents, leveraging Bayesian networks (BN) and the N-K model. Firstly, text mining technology and fault tree analysis are employed to analyze the causes of crane accidents and categorize the associate risk factors. Secondly, the types of risk coupling resulting from human, machine, management, and environmental risk factors are defined. Thirdly, the BN model is developed based on the analysis of crane accident risksand its N-K model. Fourthly, the parameters of the risk coupling nodes in the developed BN are determined based on the calculation results of the N-K model. Finally, for the risk coupling types with high coupling values and the first-level node and second-level node, the failure probability is analyzed through posterior probability and sensitivity analysis. The results indicate that factors related to man and management significantly impact crane accidents and warrant enhanced attention. The interplay among multiple risk factors significantly influences the probability of crane accidents, necessitating careful attention.

## Introduction

As one of the most critical means of vertical lifting and horizontally moving materials, crane operations are widely used in infrastructure construction and other fields^1^. During crane operations, the complex man–machine-management-environment interface, high task difficulty, and uncertain information^2^, contibuite to a potential high risk, leading to the causes of crane accidents. These causes, encompassing overload, collisions, and operation errors, are highly complex and can easily results in crane accidents, leading to serious consequences such as casualties and property losses^3^. Therefore, research the man–machine–management–enviroment interface of crane accidents holds significant meaning for preventing the occurrence of lifting injury accidents. Additionally, ensuring the safety of lifting operations and reducing economic losses are important issues that urgently need resolution in the construction of large-scale water conservancy and hydropower projects, long-span bridges, high-rise buildings, and other projects^4,5^.

However, as a critical facility and equipment associated with national economic construction and societal functioning, lifting machinery exhibits characteristics of high technical complexity, stringent operational requirements, challendingt maintenance, and elevated potential risks^6^. Various safety risks fromdiverse factors, inluding human, machanical, environmental, and managerial aspects, during crane operation. The safety state of lifting operations flucates with alterations in human factors, material factors, environmental factors, and management factors. It can be observed that the interaction among risk factors in lifting accidents exhibits high-order nonlinearity^7^, and the complexity of the risk coupling relationshipheightens the inteicacy and challenges associated withe safety control of lifting operations, rendering it highly unfavorable for effective risk management. Notably, the coupling among risks serves as the primary driving force for risk evolution. If the risk value resulting from coupling surpasses the safety threshold, it could result in a lifting accident. For instance, the factors contributing to the collapse of the gantry crane at the wharf site of Hudong Zhonghua Shipyard Co., Ltd. encompass improper safety supervision, illegal worker operation, low safety awareness, and improper investigation of safety hazards. The disaster resulted in a total of 36 deaths, 3 injuries, and direct economic losses amounting to nearly 80 million RMB. Therefore, disregarding the coupling effect among different risk factors, adapting a linear perspective, or inadequately considering risk occurrence will result in deviations in the estimating actual risk threats and losses^8^.

To mitigate crane accidents, numerious scholars have validated various accident prevention methods.These methods encompass the utilization of frequency and complex network topology parameters, aiming to identify the key causes of crane accidents^9,10^. Many scholars have conducted extensive studies on accident prevention. Extensive research has been conducted on the safety risks of lifting operations, considering various perspectives, including people, machinery, environment, and organization at the lifting site^11–14^. The installation and disassembly of cranes are highly perilous, constituting the primary stages that lead to lifting accidents and serve as focal points for safety management and control of lifting operations^15^. Additionally, Chen et al.^16^ conducted eye movement experiments focusing on tower crane operations to analyze the visual effects and optical mechanisms influencing crane drivers. However, the majority of scholars have given inadequate consideration to the coupling relationship among risk factors in crane accidents, and the interplay among different risk factors significantly influendes the occurrence, development, and severity of crane accidents^17^. However, most of these conventional research methods rely on expert experience to investigate the causes of lifting accidents, leading to subjective results. The N-K model can capture the coupling of accident causes through analysis of existing accident reports. Subsequently quantitative research can be conducted. The BN network can serve as an effective tool for inferring the coupling relationship among various factors. Therefore, this study will explore the coupling among the causes of lifting accidents by integrating the N-K model and the BN model.

This paper introduces a new method to quantify the coupling of accident causes using both the BN and the N-K models. This paper unveils the risk coupling mechanism of crane accidents, examing interactions among various risk factors. The method proposed is described in "Materials and methods". "Model construction" presents a detailed description of the model construction process, encompassing model structure definitions and parameter specifications. "Results and discussions" is covers the results and discussion. "Conclusion" provides a summary of the paper.

## Materials and methods

### The proposed method

**Figure 1:**
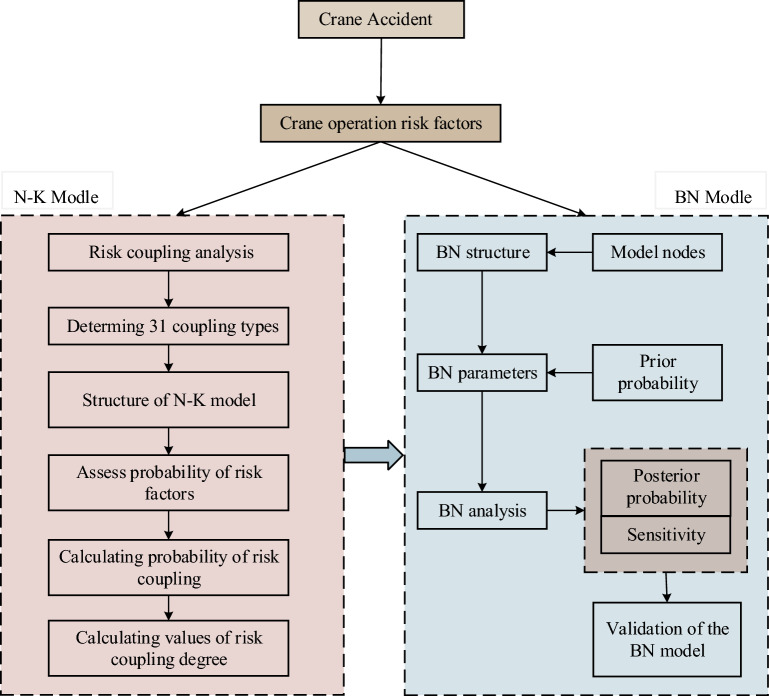
The proposed method.

The BN structure is established through the analysis of risk factors using text mining technology and the N-K modelstructure. The proposed method is illustated in Fig. 1. To determine the structure of the N-K model, it is imperative to identify the type of risk coupling. Leveraging expert experience and crane accident reports, text mining technology is employed to identify risk factors and their probability. Subsequently, the values of various risk coupling degrees are calculated using the N-K algorithm. These data are incorporated into the BN model to define the parameters, and posterior probability and sensitivity analyses are conducted, respectively.

This method can delineate the coupling relationship among risk factors in various subsystems, quantitatively analyze the uncertainty and the effect of risk coupling, demonstrate the dynamic characteristics of risk evolution and risk coupling, and analyze how risk factors contribute to the evolution process of triggering accidents. The research findings can serves as references for enhancing the safety control of lifting operations.

### Bayesian network of the risk factors in crane operation

A Bayesian Network (BN) is a graphical network rooted in probabilistic reasoning, comprising nodes and directed edges. Nodes in the network represent variables, and the directed edges dipict interaction among these nodes^18^. It can effectively achieve the representation of uncertain knowledge and support complex reasoning. In this context, the risk factors associated with crane operations are assigned to nodes, and the coupling relationship among different risk factors are represented by edges, culminating in the establishment of the Bayesian network model of the risk factors of crane operations^19,20^.

### N-K model of the risk factors in crane accidents

The N-K model was initially introduced by Kauffman and Weinberger^21^ within the realm of evolutionary biology and subsequently explored further by Kauffman^22^. It aims to delineate the roles of interdependence and complexity in complex adaptive systems. It is extensively utilized for the degree of correlation and risk coupling among factors contributing to unsafe events. The N-K model comprises two parameters: *N* denotes the number of elements, and *K* represents the number of coupling relationships (as obtained by consulting experts in related fields). In a complex system comprising n elements, where each element has n states, then nN possible couplings.

By quantifying the interactive information coupling among risk factors in crane operations, the risk factors of four subsystems: Man, Machine, Management, and Environment are effectively measured, leading to the formation of a new risk state. The formula for calculating interactive information is as follows:

1 $$R(M_{1} ,M_{2} ,M_{3} ,E) = \sum\limits_{{m_{1} = 1}}^{{M_{1} }} {\sum\limits_{{m_{2} = 1}}^{{M_{2} }} {\sum\limits_{{m_{3} = 1}}^{{M_{3} }} {\sum\limits_{e = 1}^{E} {P_{{m_{1} m_{2} m_{3} e}} } } } } \times \log_{2} \left[ {P_{{m_{1} m_{2} m_{3} e}} /P_{{m_{1} ...}} \times P_{{.m_{2} ..}} \times P_{{..m_{3} .}} \times P_{...e} } \right]$$

where *R*_M1M2M3E_ represents the probability associate with Man being in state m_1_, Machine being in state m_2_, Management being in state m_3_, Environment being in state e while the four factors are coupled. The variables are defined as following: *m*_1_ = 1,2,…,M_1_; *m*_2_ = 1,2,…,M_2_; *m*_3_ = 1,2,…,M_3_; *e* = 1,2,…,E. Furthermore, *R*_m1_, *R*_m2_, *R*_m3_, and *R*_e_ are the probabilities of each risk factor in different states. The *R* value as a comprehensive measure of the coupling effect of safety risks in lifting operations, where a higher *R* value corresponds to an increased probability of a lifting operation occurrence.

## Model construction

### The definition of crane operation risk factors

**Figure 2:**
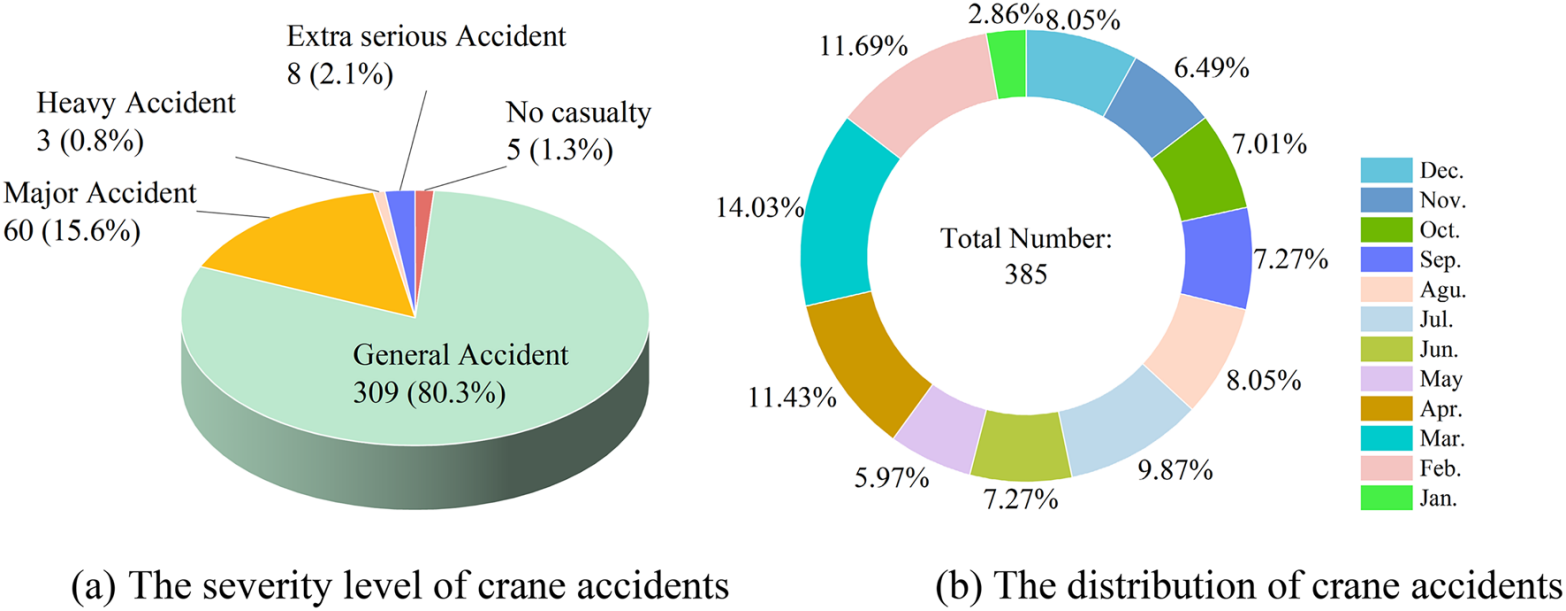
The situation of crane accident.

Firstly, nearly 400 valid crane accident reports spanning the years 2000 to 2021 were systematically crawled. These reports encompass crucial datails, including the fundamental accident scenario, causal factors, identification of responsibility, preventive measures, and recommendations rectification. Moreover, the reported incidents are substantiated as accurate and reliable, covering diverse provinces and cities across China, rendering the data suitable for research purposes. The severity of each incident is assessed, distinguishing those without personal injuries from the general accident category and labeling them as "no casualty accidents," as illustrated in Fig. 2a. Based on this dataset of crane accidents, the monthly frequency of incidents is tabulated according to their occurrence, as depicted in Fig. 2b.

As depicted in Fig. 2a, there were five accidents without casualties, constituting 1.3% of the tatal. Despite the absence of personal casualties in these incidents, they frequently resulted in substantial economic losses. Additionally, there were 309 instances of general accidents, representing 80.3%, and 60 major accidents, accounting for 15.6%. This suggests that the overall impact of lifting injury accidents was relatively limited, and in some instances, there were a few casualties. Specifically, there were eight major accidents, constituting 2.1%, and three major accidents, constituting 0.8%.

Observing Fig. 2b, it is evident that the lowest number of accidents occurs in January, and fewer accidents are reported in May–June and August-November, indicating favorable conditions for crane operations. Conversely, the hightest number of accidents is observed in February-April, and July and December register the highest frequency of accidents. These patterns are associated with management factors, including safety management inspection time and construction weather in China^23^.

**Figure 3:**
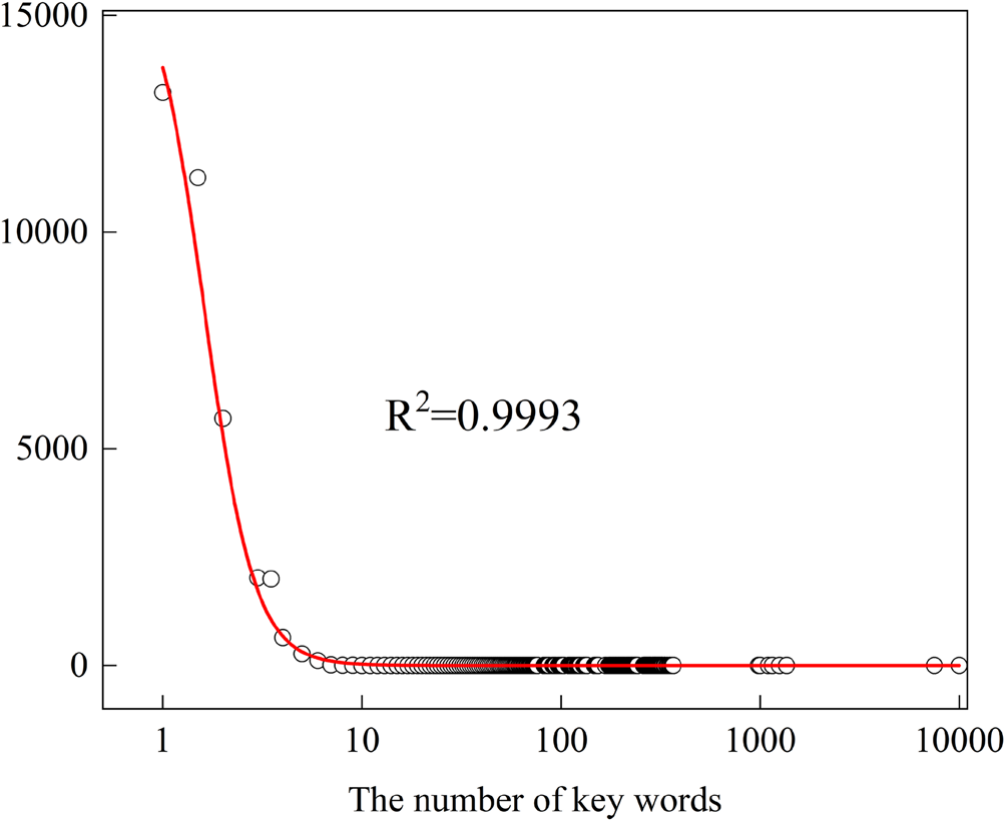
Word frequency fitting trend of crane accidents.

In this study, 385 crane accident reports are compiled and processed through text mining technology. Applying system safety theory, crane operation is analyzed as a word frequency statistic across four subsystems: Man (*M*_1_: crane driver, on-site construction worker, commander, safety supervisor), Machine (*M*_2_: crane), Management (*M*_3_: lifting operation safety regulations, on-site safety supervision measures, etc.), and Environment (E: crane operation surrounding environment, weather conditions, etc.). High-frequency words are fitted, and the results, illustrated in Fig. 3 below, demonstrate a well-fitting effect with the fitting coefficient *R* = 0.9967.

As depicted in Fig. 3, the majority of words have a low frequencies, with only a small number appearing at high frequencies. The range of word types is limited, suggesting that a restricted number of high-frequency words can more effectively capture crucial information about the causes of heavy injury accidents.

**Figure 4:**
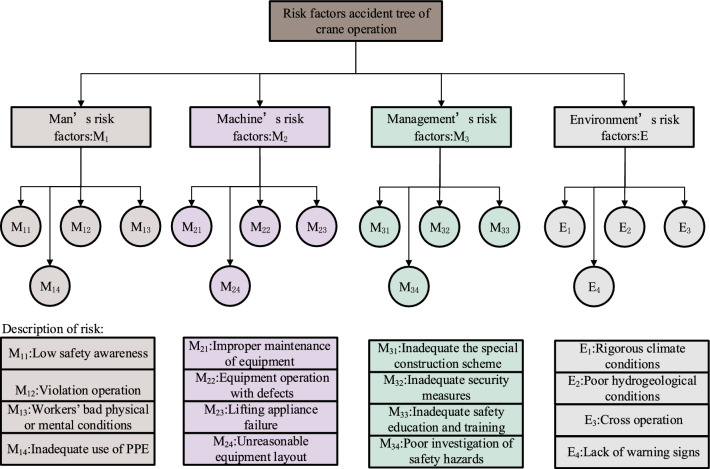
Fault tree of crane accidents.

The fault tree method can efficiently analyze the direct causes and uncover potential causes of accidents, contributing to the valid identification of risks within the crane operation system^24^. The fault tree method, along with the statistical hazard source method, is emplyed to establish the fault tree for crane accident safety risks, as illustrated in Fig. 4.

### Risk coupling mechanism of crane accidents

**Figure 5:**
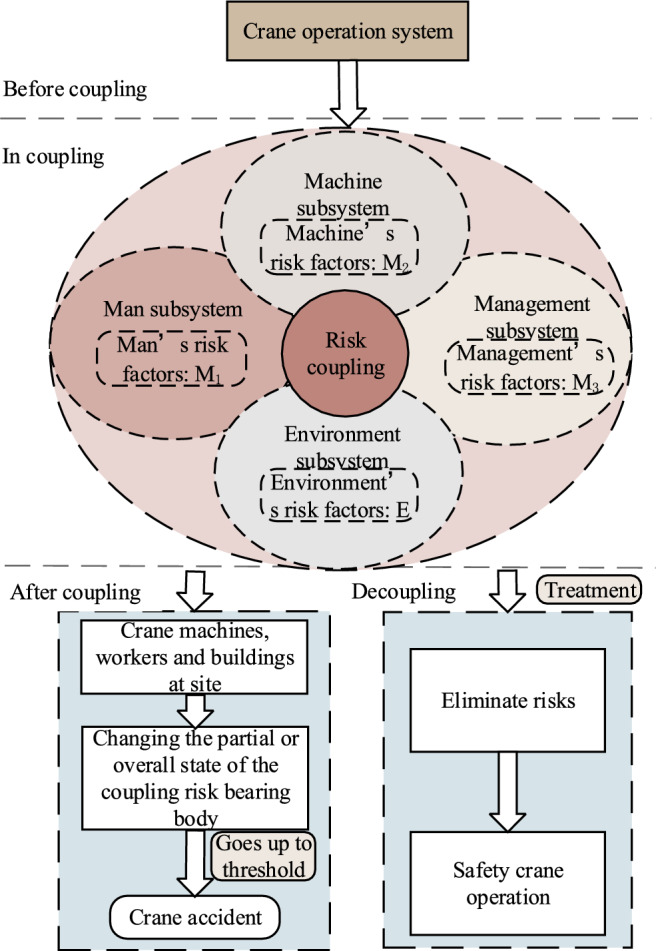
Risk coupling mechanism of crane accidents.

The crane machinery, field personnel, and surrounding buildings are defined as coupling risk bearers of crane accidents^25^, subject to direct or indirect effects from various risk factors within the four subsystems. If the coupling effect of these risk factors surpasses a predefined safety threshold, it results in a lifting accident and inflicts damage upon each risk bearer^26,27^. The coupling effect among the various subsystems is shown in Fig. 5.

### Risk coupling styles of crane accidents

**Figure 6:**
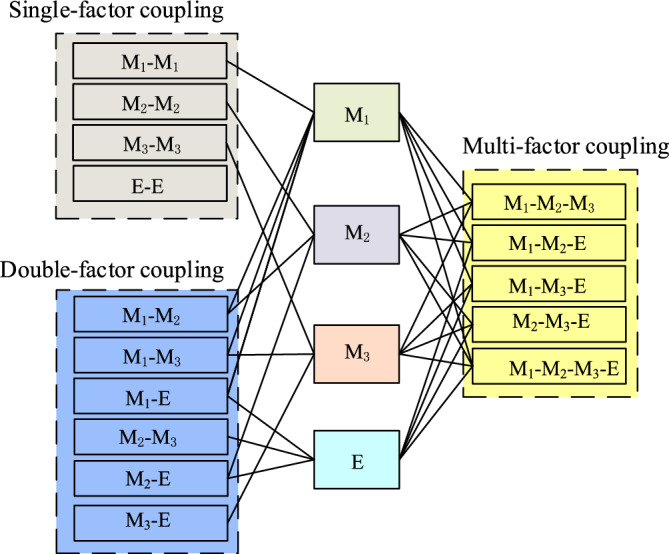
Risk coupling styles of crane accidents.

Risk coupling among different subsystems of the crane operation system is categorized into three categories^28–30^: single-factor coupling, double-factor coupling, and multi-factor coupling with the corresponding coupling styles are shown in Fig. 6.

Single-factor risk coupling pertains to the coupling of risk factors within each of the four subsystem, Machine, Management, and Environment. There are four types of risk factors, namely, *M*_1_–*M*_1_ coupling, *M*_2_–*M*_2_ coupling, *E*–*E* coupling, and *M*_3_–*M*_3_ coupling The coupling risk values are denoted as *R*_11_, *R*_12_, *R*_13_, and *R*_14_, respectively. The total coupling degree value is recorded as *R*_1_, but in cases by a risk factor, so there is no risk coupling, which is a special case.

Double-factor risk coupling involves the coupling of risk factors in two distinct subsystems among the four subsystems: Man, Machine, Management, and Environment. This includes *M*_1_–*M*_2_ coupling, *M*_1_–*M*_3_ coupling, *M*_1_–*E* coupling, *M*_2_–*M*_3_ coupling, *M*_2_–*E* coupling, and *M*_3_–*E* coupling, amonting to a total of six categories. The respective coupling risk values are recorded as *R*_21_, *R*_22_, *R*_23_, *R*_24_, *R*_25_, and *R*_26_, with the total coupling degree value is recorded as *R*_2_.

Multi-factor risk coupling involves the coupling of risk factors acorss three or four different subsystems among the four: Man, Machine, Management, and Environment. This includs *M*_1_–*M*_2_–*M*_3_ coupling, *M*_1_–*M*_2_–*E* coupling, *M*_1_–*M*_3_–*E* coupling, *M*_2_–*M*_3_–*E* coupling, and *M*_1_-*M*_2_-*M*_3_-*E* coupling, for a total of five kinds. The coupling risk values are recorded as *R*_31_, *R*_32_, *R*_33_, *R*_34_, and *R*_41_, and the total coupling degree value of multi-risk factors is recorded as *R*_T_.

### Calculation of coupling effect of risk factors

The safety risk in crane operations arises from the localized interaction of risk-coupling following the coupling of various risk factors. This can be effectively assessed by the calculating the effects of single-factor coupling, double-factor coupling and multi-factor coupling effects in crane operation risk factors. Leveraging a wealth of accident reports, a layered decomposition is conducted, and the frequency of decomposition for each risk factor (Man, Machine, Management, and Environment) is tallied. Risk factors in a safe state are recorded as '0', where those in a dangerous state leading to accidents are recorded as '1'. The 385 crane accidents occurring from 2000 to 2021 are divided in detail, and the occurrences of single, double, and multi-factor coupling fresulting from the coupling of risk factors in each subsystem are counted. The results are shown in Table 1.

**Table 1:** Coupling styles number of crane accidents.

Coupling style	Number	Coupling style	Number	Coupling style	Number
1111	54	1100	27	0011	23
1000	15	1010	33	1110	21
0100	17	1001	13	1011	33
0010	21	0110	25	1101	38
0001	7	0101	22	0111	36

Considering the type of risk coupling, the calculation formulas of 6 types of two-factor risk coupling are given by formula (2), while the calculation formulas of 5 types of multi-factor risk coupling are provided by formula (3). The resulting settlement are as follows. Additionally, the coupling degree value for single factor is too small, so it is not included in the table^31^.

2 $$\begin{gathered} R_{2} = \left\{ \begin{gathered} R_{21} = \sum\limits_{{m_{1} = 1}}^{{M_{1} }} {\sum\limits_{{m_{2} = 1}}^{{M_{2} }} {P_{{m_{1} m_{2} }} } } \times \log_{2} \left[ {P_{{m_{1} m_{2} }} /P_{{m_{1} ...}} \times P_{{.m_{2} ..}} } \right] \hfill \\ R_{22} = \sum\limits_{{m_{1} = 1}}^{{M_{1} }} {\sum\limits_{{m_{3} = 1}}^{{M_{3} }} {P_{{m_{1} m_{3} }} } } \times \log_{2} \left[ {P_{{m_{1} m_{3} }} /P_{{m_{1} ...}} \times P_{{..m_{3} .}} } \right] \hfill \\ R_{23} = \sum\limits_{{m_{1} = 1}}^{{M_{1} }} {\sum\limits_{e = 1}^{E} {P_{{m_{1} e}} } } \times \log_{2} \left[ {P_{{m_{1} e}} /P_{{m_{1} ...}} \times P_{...e} } \right] \hfill \\ R_{24} = \sum\limits_{{m_{2} = 1}}^{{M_{2} }} {\sum\limits_{{m_{3} = 1}}^{{M_{3} }} {P_{{m_{2} m_{3} }} } } \times \log_{2} \left[ {P_{{m_{2} m_{3} }} /P_{{.m_{2} ..}} \times P_{{..m_{3} .}} } \right] \hfill \\ R_{25} = \sum\limits_{{m_{2} = 1}}^{{M_{2} }} {\sum\limits_{e = 1}^{E} {P_{{m_{2} e}} } } \times \log_{2} \left[ {P_{{m_{2} e}} /P_{{.m_{2} ..}} \times P_{...e} } \right] \hfill \\ R_{26} = \sum\limits_{{m_{3} = 1}}^{{M_{3} }} {\sum\limits_{e = 1}^{E} {P_{{m_{3} e}} } } \times \log_{2} \left[ {P_{{m_{3} e}} /P_{{..m_{3} .}} \times P_{...e} } \right] \hfill \\ \end{gathered} \right. \hfill \\ \hfill \\ \end{gathered}$$

3 $$\begin{gathered} R_{T} = \left\{ \begin{gathered} R_{31} = \sum\limits_{{m_{1} = 1}}^{{M_{1} }} {\sum\limits_{{m_{2} = 1}}^{{M_{2} }} {\sum\limits_{{m_{3} = 1}}^{{M_{3} }} {P_{{m_{1} m_{2} m_{3} .}} } } } \times \log_{2} \left[ {P_{{m_{1} m_{2} m_{3} .}} /P_{{m_{1} ...}} \times P_{{.m_{2} ..}} \times P_{{..m_{3} .}} } \right] \hfill \\ R_{32} = \sum\limits_{{m_{1} = 1}}^{{M_{1} }} {\sum\limits_{{m_{2} = 1}}^{{M_{2} }} {\sum\limits_{e = 1}^{E} {P_{{m_{1} m_{2} .e}} } } } \times \log_{2} \left[ {P_{{m_{1} m_{2} .e}} /P_{{m_{1} ...}} \times P_{{.m_{2} ..}} \times P_{...e} } \right] \hfill \\ R_{33} = \sum\limits_{{m_{1} = 1}}^{{M_{1} }} {\sum\limits_{{m_{3} = 1}}^{{M_{3} }} {\sum\limits_{e = 1}^{E} {P_{{m_{1} .m_{3} e}} } } } \times \log_{2} \left[ {P_{{m_{1} .m_{3} e}} /P_{{m_{1} ...}} \times P_{{..m_{3} .}} \times P_{...e} } \right] \hfill \\ R_{34} = \sum\limits_{{m_{2} = 1}}^{{M_{2} }} {\sum\limits_{{m_{3} = 1}}^{{M_{3} }} {\sum\limits_{e = 1}^{E} {P_{{.m_{2} m_{3} e}} } } } \times \log_{2} \left[ {P_{{.m_{2} m_{3} e}} /P_{{.m_{2} ..}} \times P_{{..m_{3} .}} \times P_{...e} } \right] \hfill \\ R_{41} = \sum\limits_{{m_{1} = 1}}^{{M_{1} }} {\sum\limits_{{m_{2} = 1}}^{{M_{2} }} {\sum\limits_{{m_{3} = 1}}^{{M_{3} }} {\sum\limits_{e = 1}^{E} {P_{{m_{1} m_{2} m_{3} e}} } } } } \times \log_{2} \left[ {P_{{m_{1} m_{2} m_{3} e}} /P_{{m_{1} ...}} \times P_{{.m_{2} ..}} \times P_{{..m_{3} .}} \times P_{...e} } \right] \hfill \\ \end{gathered} \right. \hfill \\ \hfill \\ \end{gathered}$$

**Figure 7:**
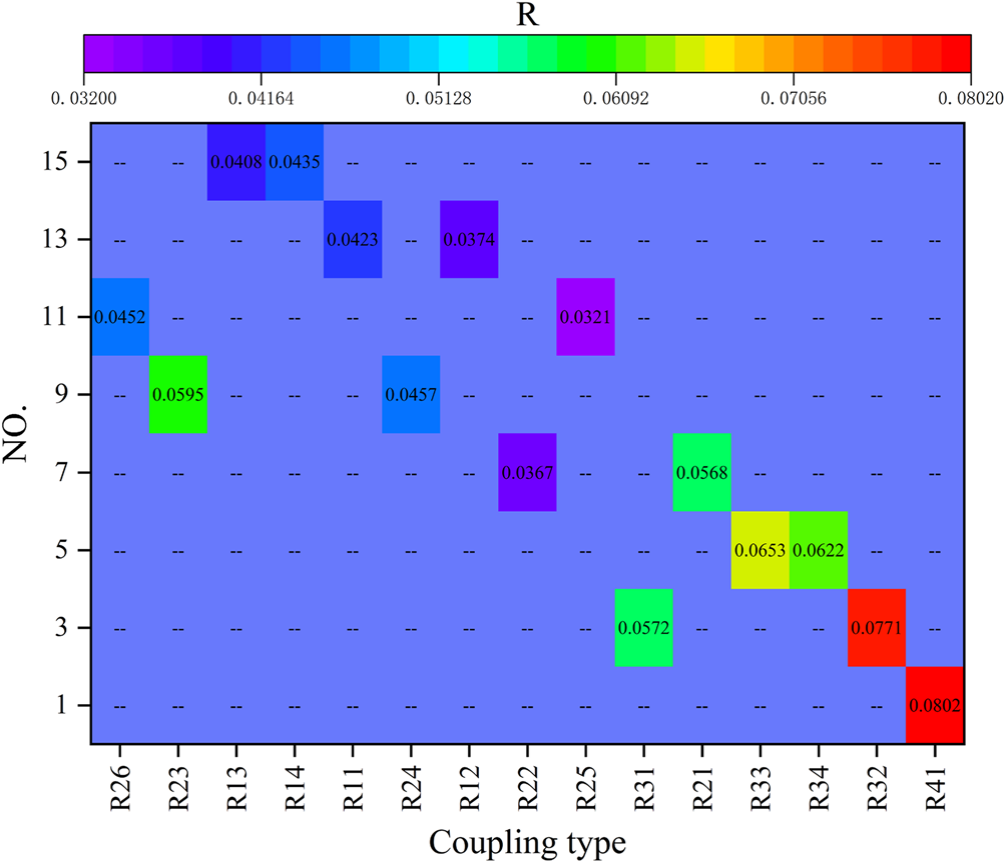
Risk factors coupling probability.

Utilizing Eqs. (2) and (3), the coupling degree values of risk factors are computed. Observations reveal that the coupling degree value of risk factors is directly proportional to the number of risk factorsinvolved in coupling, with the order *R*_4_ > *R*_3_ > *R*_2_ > *R*_1_. The coupling degree value of 4 factors is the highest, followed by multi-factor, double factors, and single factors. In the context of multi-factor coupling risk, the values are as follows: *R*(*M*_1_, *M*_2_, *M*_3_, *E*) = 0.0802, *R*(*M*_1_, *M*_2_, *M*_3_) = 0.0746, and *R*(*M*_1_, *M*_3_, *E*) = 0.0690. The risk factors of Man and Management are included in the larger risk coupling degree value, indicating that the risk of Man and Management is most easily coupled with other risks, which poses a threat to the safety system of crane operations. In addition, in the multi-factor coupling, the risk factors of the Management are included in the risk coupling degree value, which manifests that the lack of safety education and training and the special construction plan are the main causes of crane accidents (Fig. 7).

### The development of the BN model

**Figure 8:**
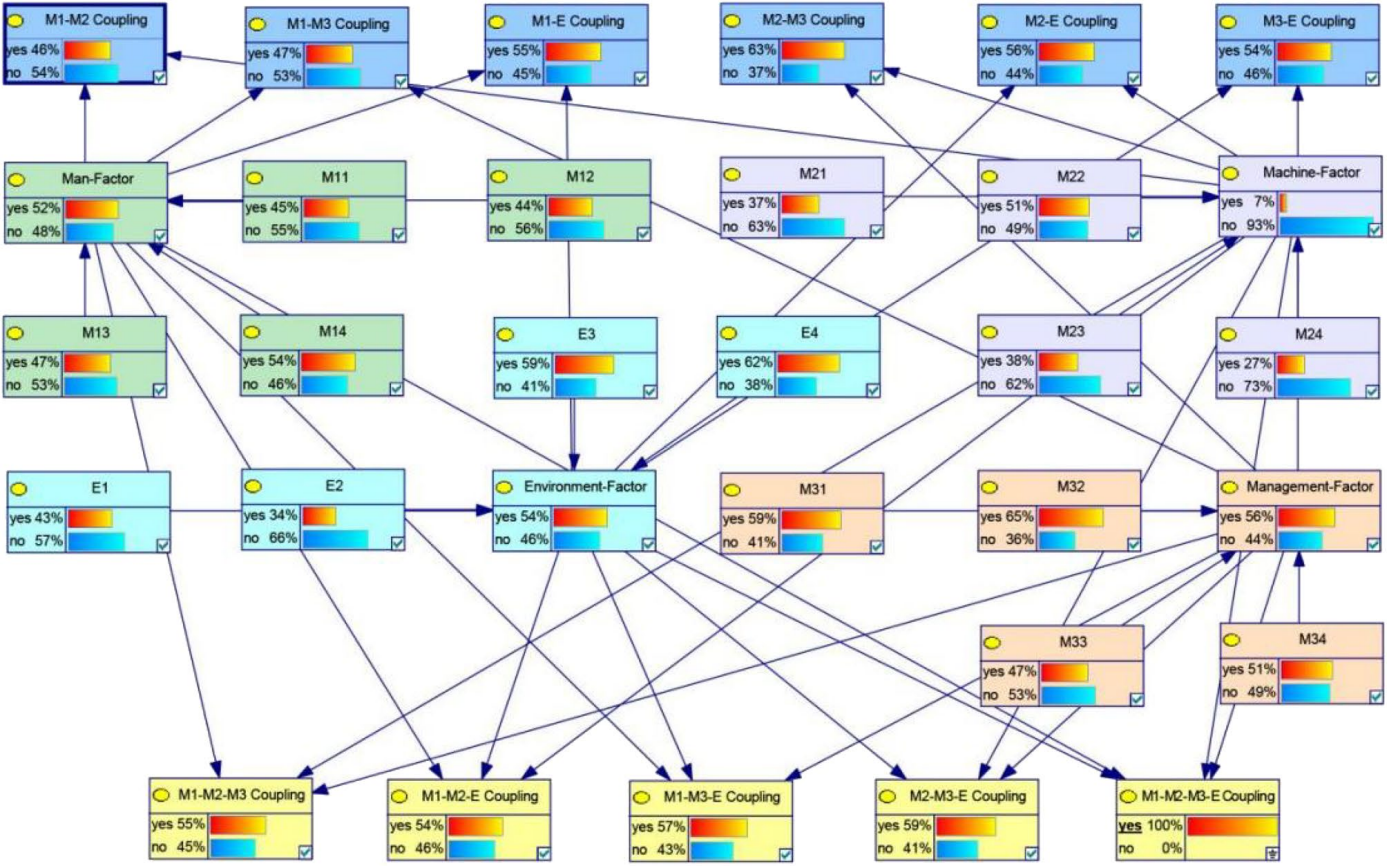
Expanded BN based on the N-Kmodel.

The Bayesian Network(BN) structure depicting crane accidents is illustrated in Fig. 8. In this study, Genie software is employed to develop the BN model. Based on the 16 risk factors identified in "The definition of crane operation risk factors", the node variables of the BN model can be preliminarily established. Assigning two states, whether the nodes exist or not, represents the occurrence or non-occurrence of the state described by the node. The construction of the BN model under factors coupling involves two steps: (1) Determine whether there are edges between nodes according to the results of the N-K model calculation; (2) considering the causal relationship among nodes, the arrow direction of each edge is determined to form a complete BN topology.

### Validation of the developed BN model

Model verification constitutes a crucial aspect of the proposed model. The three-kilometer verification method, a common common approach for Bayesian networks, is employed in this study to assess the effectiveness of the proposed model. The three-kilometer theory should adhere the following criteria: (1) a change in the prior subjective probability of each parent node should result in a change in the posterior probability of the child node; (2) the change of the subjective probability of each parent node has the same influence on the value of the child node; (3) the change of attribute probability of x–y (y ϵ x) has less influence on the value than x^32^.

When the vibration of the parent node of the child node Man-Factor is set to 0, the probability of M1–M3 coupling decreases from 47 to 23%. If the low safety awareness is set to 0, the probability of M1–M3 coupling will decrease to 9.2%. If all the parent nodes are set to 0, the probability of M1–M3 coupling is also 0. Repetitive training of these nodes to conform to the axioms enables model validation.

## Results and discussions

The reasoning ability of a BN model denotes the capability to manipulate the nodes in the network to deduce changes in other nodes based on the established Bayesian network. The probability of occurrence or non-occurrence of any node can be calculated using the method of conditional probability in the Bayesian network. Furthermore, any alteration in the input value of any node may lead to a change in the probability value of the target node.

Based on the established Bayesian network depicting the coupling of crane accident risk factors, sensitivity analysis and the analysis of the most approximate cause chaina are conducted for the three risk factors *M*_1_–*M*_2_-*M*_3_–*E*, *M*_1_–*M*_2_–*M*_3_, and *M*_1_–*M*_3_–*E.* This exploration identifies the sensitivity risks and key factors in risk coupling, offering a scientific foundation for the safety management of crane operations.

### Prior probability and posterior probability

**Figure 9:**
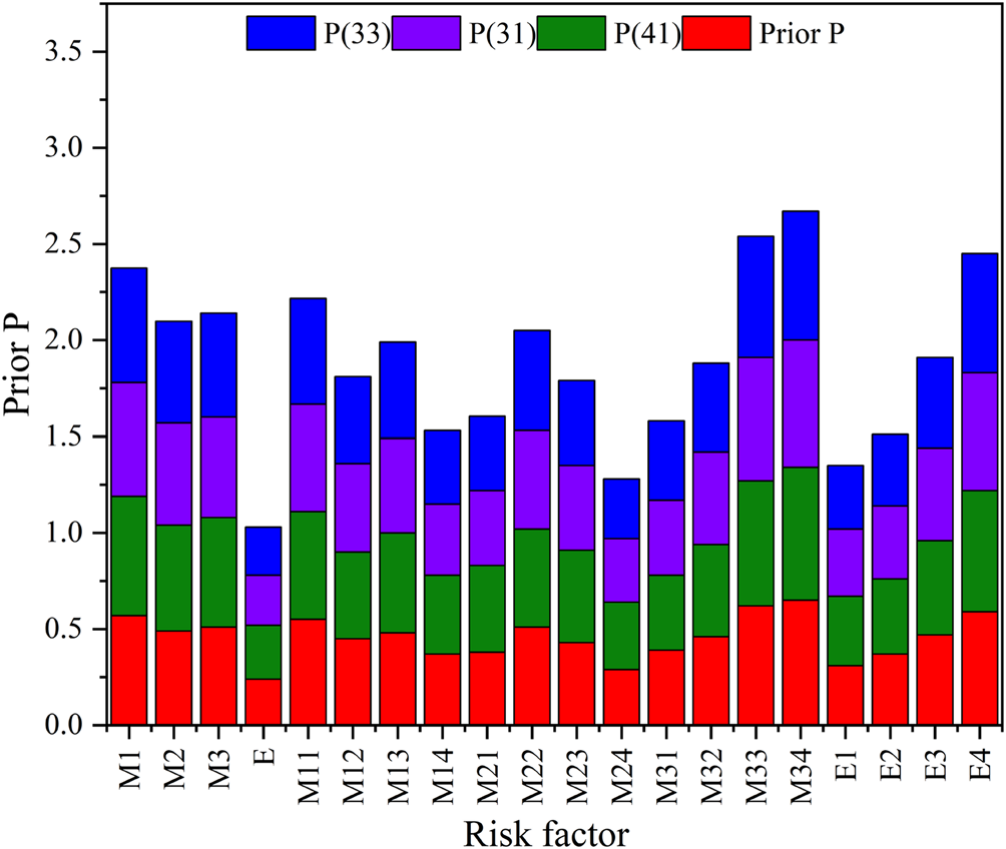
Failure probability distribution of risk factor.

A comparative analysis of the prior probability and posterior probability results for each node in the above three high-risk coupling effects is presented in Fig. 9.

From the posterior probability, it can be inferred that the risk factor *M*_3_ of the *M*_1_–*M*_2_–*M*_3_–*E* coupling type is most significant, constituting 69.1%, making it the primary factor contributing to the lifting operation accidents. Environmental factors exert the least influence on the risk of coupling in crane operations. Illegal operations emerge as a important factor leading to lifting accidents.

### Sensitivity analysis

**Figure 10:**
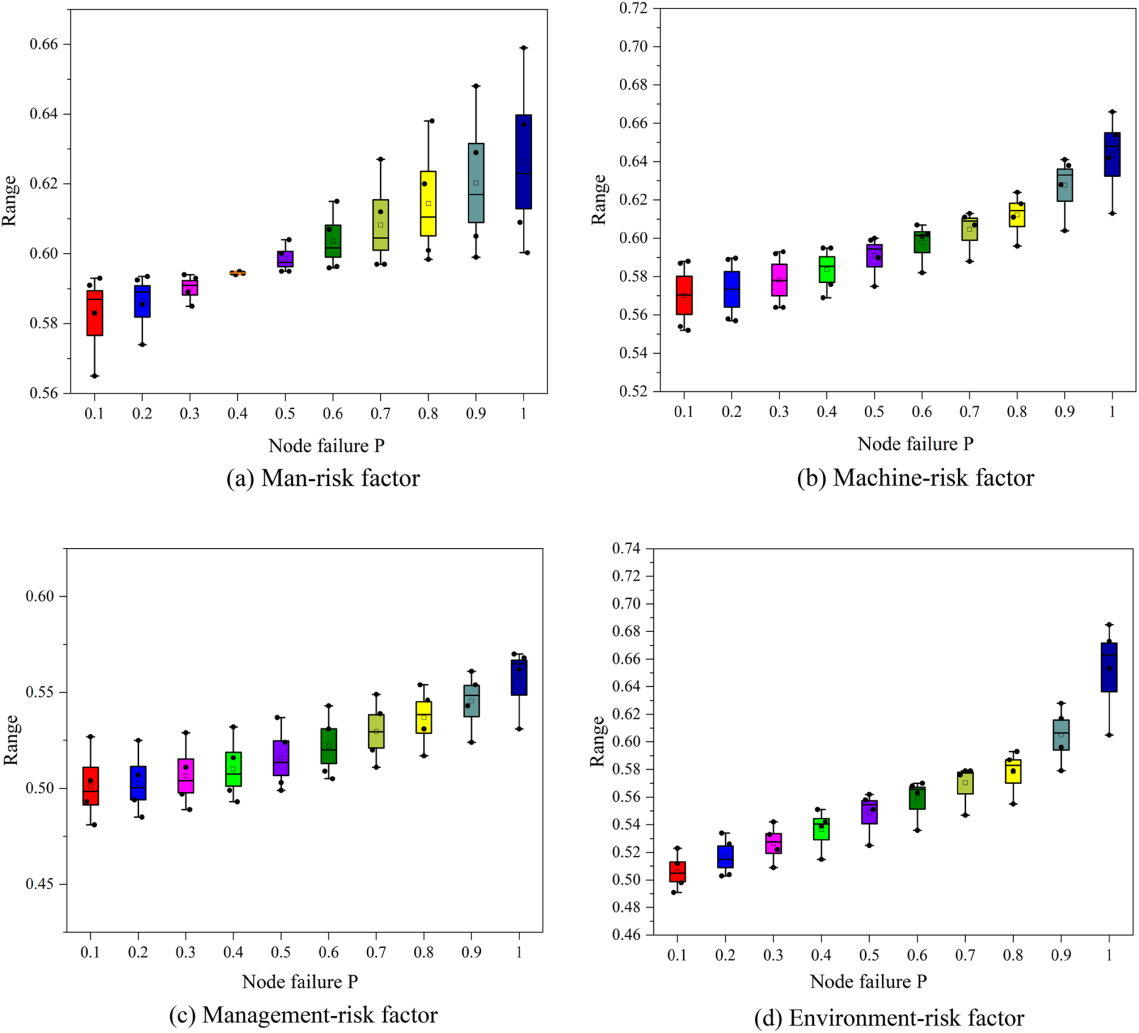
Curve of first-order node probability.

The BN model suggests that the impact of the change in each factor on the risk coupling of crane operations varies.For the analysis of the influence trend of the value change of each factor on the risk coupling, the factors are adjusted within a certain range during the selection of uncertain factors in the sensitivity analysis, such as ± 5%, ± 10%, ± 15%, ± 20%, etc. Consideration must be given to the smoothness of the curve when choosing the specific step size. If the step size is too large, the curve may lack smoothness. Contraryly, a very small step size will result in a large calculation load and excessive smoothness. In the analysis of sensitivity for the *M*_1_–*M*_2_–*M*_3_–*E* with the largest risk coupling degree value, the factor value step size is 0.1, and the value range is [0, 1]. Subsequently, the probability sensitivity of related nodes affected by single-factors of Man, Machine, Environment, and Management is analyzed, and the key nodes of BN probability change are identified. When the probability of each node is 0, the probability value of other nodes does not change; that is, the risk coupling of crane operations is not affected by this factor. The abscissa is the probability value of node failure, and the ordinate is the probability of lifting accidents caused by multi-factor risk coupling. The results are shown in Fig. 10.

Figure 10a illustrate that as the failure probability of the nodes related to Man risk factors increases from 0 to 1, the impact of each node on the crane accident grows, with a substantial change at the end. This suggests that Man risk factors have a significant impact on the crane accident. Specifically, low safety awareness and illegal operations exert a greater impact on the probability of crane accidents. In Fig. 10a, c, the box type rises steadily, indicating that the risk factors of the Management and Machine have a significant and stable impact on the crane accident; in Fig. 10d, it maintains steady growth in the early stage but increases sharply, with a notable difference when the probability is 0.9. This implies that the failure probability of environmental factors increases to a certain extent, exerting a significant impact on crane accidents^33^.

**Figure 11:**
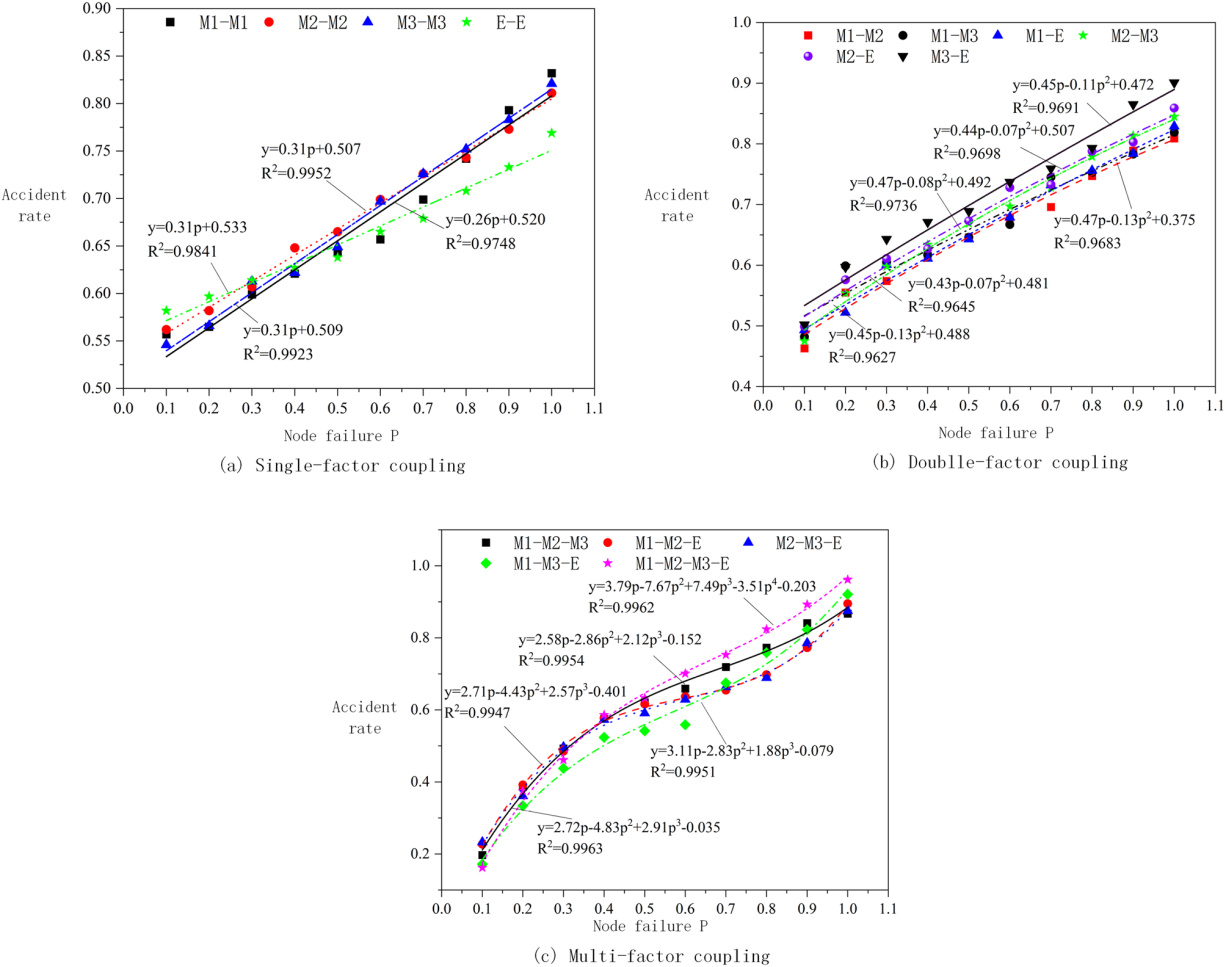
Probability curve for secondary nodes.

To analyze the influence trend of single-factor, double-factor, and multi-factor on the coupling effect of crane operations, the probability changes of Man, Machine, Management, and Environment secondary nodes are drawn in Fig. 11.

Figure 11a implies that the single-factors of Man, Machine, Management, and Environment exhabit a linear positive correlation with the risk of crane operation accidents, and the fitting correlation coefficient *R*^2^ is greater than 0.98. The risk of crane operation accidents increases with the increase in the failure probability value of single-factor nodes, and the influence of Management factors is the most significant. Observing Fig. 11b, it is evident that the double-factor coupling curve of Man, Machine, Management, and Environment forms a quadratic positive correlation function, with the correlation coefficient *R*^2^ exceeding 0.96. The risk of a crane accident increases with the increase in double-factor failure probability, and the change range of the Man–Machine factor is the largest, at 98.07%. The probability change and fitting curve of a crane operation accident under the combined action of Man, Machine, Management, and Environment are shown in Fig. 11c. The fitting curve is positively correlated, and the fitting accuracy *R*^2^ is greater than 0.99. At a failure probability of 100%, the error probability reaches as high as 94%. Hence, to prevent the occurrence of crane accidents, attention should be paid to reducing the impact of multi-factors on crane operations at the same time^34^.

## Conclusion

This paper presents a methodology for quantifying the risk coupling of crane accidents using Bayesian Network and the NK models. The paper defines the types of risk coupling caused by Man factors, Machine factors, Management factors and Environment factors in crane accidents. The paper unveil the risk coupling mechanism of crane accidents by examing the interactions among different types of risk factors.

The BN structure is established based on the risk analysis of crane accidents and its N-K model. The parameters of risk coupling nodes in the developed BN are determined by the calculation results from the NK model. Following the completion of the BN model, validation is carried out using posterior probability, and further analysis, including sensitivity analysis and uncertainty analysis, are conducted to investigate the effects of risk factors on different types of risk coupling.

Through reasoning simulation analysis, it is determianed that among the influencing factors at the root nodes, workers' illegal operations, inadequate safety supervision, and bad weather conditions are high-risk factors that induce accidents and should be paid more attention to in risk prevention and control. Among the influencing factors of the first-level node, the failure probability caused by Man factor failure and Management factor failure changes more greatly, and the training management and on-site supervision of construction workers should be strengthened. Among the influencing factors of the secondary node, the crane accident increases in the order of single-factor, double-factor, and multi-factor, and the double-factor and multi-factor cases are nonlinear.

This paper expands the theoretical approach to analyzing safety risk factors in lifting operations, offering a theoretical reference for establishing a dual prevention mechanism for safety accident risks in lifting operations. It also enriches the theoretical methods for the prevention and control of safety accident risks in construction projects. It provides guidance for formulating schemes, including hazard source identification, accident prevention in advance, accident rescue in the event, and accident investigation after the event.

Each risk factor in the safety accident of lifting operations will change over time, and the risk coupling effect at different time nodes will also vary. Therefore, future research will consider the influence of time parameters and establish a dynamic analysis model of the risk coupling effect of safety accidents in lifting operation.

## Data Availability

The datasets generated and/or analysed during the current study are not publicly available and are available from the corresponding author on reasonable request.
